# Development and validation of a short version of the Female Sexual Function Index in the Spanish population

**DOI:** 10.1186/s12905-021-01213-8

**Published:** 2021-02-11

**Authors:** Laura Mateu Arrom, Montserrat Girabent-Farrés, Mónica González, Joan Palou, Carlos Errando-Smet, Inés Ramírez-García

**Affiliations:** 1 Functional and Female Urology Department, Fundació Puigvert, C/Cartagena 340-350, 08025 Barcelona, Spain; 2 Department of Physioterapy, School of Health Sciences, TecnoCampus-Pompeu Fabra University, C/Mallorca, 198 4º2ªD, 08036 Mataró, Barcelona Spain; 3 Andrology Department, Fundació Puigvert, C/Cartagena 340-350, 08025 Barcelona, Spain; 4 Urology Department, Fundació Puigvert, C/Cartagena 340-350, 08025 Barcelona, Spain; 5 Functional and Female Urology department, Fundació Puigvert, C/Cartagena 340-350, 08025 Barcelona, Spain; 6 Department of Physioterapy, Faculty of Health Sciences, Blanquerna - Ramon Llull University, C/Padilla 326, 08025 Barcleona, Spain

**Keywords:** Female sexual function index, Spanish FSFI, Validity and reliability, Scale, Sexual dysfunction

## Abstract

**Background:**

The Female Sexual Function Index (FSFI) is a commonly used scale for the assessment of female sexual function. Our aim was to develop and validate a Spanish short version of the FSFI.

**Methods:**

A parallel exploratory, sequential mixed-methods approach was used, involving 2 sites. The process consisted of 2 steps: (1) cognitive and content validation of the previously translated FSFI in the Spanish population, both through a focus group; and item selection based on the difficulty and discrimination parameters using item response theory (IRT), thereby obtaining a short version of the scale (sFSFI-sv); (2) assessment of test–retest reliability (intraclass correlation coefficient, ICC) of the sFSFI-sv. The presence or absence of a sexual disorder variable based on clinical interview was used on the Receiver Operating Characteristic (ROC) to establish the cut off point whose Area Under the Curve (AUC) based on sensibility and specificity was maximum.

**Results:**

Specific modifications of the FSFI were made according to the focus group results. 114 women were included for IRT analysis. The initial IRT model pointed to the exclusion of items 1, 2, 5, 11, 18, and 19 (S-χ^2^ p < 0.001). Items 3, 9, 11, and 14 showed the best discrimination and difficulty parameters. On the basis of the IRT and focus group results, items 1, 3, 9, 12, 16, and 17 were included in the final sFSFI-sv. sFSFI-sv showed good reliability (ICC 0.91) in a group of 93 women. A total score ≤ 18 could indicate a higher risk of sexual disorder (sensitivity: 81.0%, specificity: 73.3%).

**Conclusion:**

A focus group and the IRT analysis allowed the development of a 6-item Spanish version of the FSFI, which showed good reliability in a group of Spanish women.

## Background

Sexuality is a key component in women's quality of life and closely related to their psychosocial wellbeing [1, 2]. Female sexual dysfunction (FSD) may be present in 19%–50% of women [3] and can result from a variety of causes, including anatomical, hormonal, and neurological factors, pelvic dysfunction, medications or drug abuse, and psychological or socio-cultural factors [4].

Despite its high prevalence, FSD continues to be under-recognized and undertreated [5, 6], because few women seek help and most health care professionals do not openly ask female patients about their sexual function [4]. As sexual dysfunction is a self-reported condition, direct questioning by clinicians about sexual health is crucial in order to identify FSD appropriately [4]. However, it may not be easy to talk directly to women about their sexual lives and to decode their answers [4]. Thus, a standardized validated scale assessing the multiple domains of female sexual function is a valuable tool.

In 2000, Rosen et al. developed the Female Sexual Function Index (FSFI) scale [7], consisting of 19 items in six domains, with the twin aims of providing a tool that would allow proper assessment of the multiple domains of women's sexual function and initiating and promoting research in the field of female sexuality. The FSFI has been shown to have good psychometric properties [8, 9] and has been used to assess sexual function in women suffering from diverse medical conditions such as diabetes, cancer, and pelvic organ prolapse, as well as in women at different life stages [10].

The FSFI has been translated into several languages around the world and is an internationally recognized scale [10]. In 2004, Blümel et al. [11] conducted the Spanish translation and validated the FSFI in the Chilean population. Although the FSFI has been widely used in Spain [12, 13] suitable cultural validations in Spanish population have only recently been published [14, 15].

While the FSFI is one of the most commonly used questionnaires in Spain to assess female sexual function, it has been considered too long for use in either research or routine outpatient clinics as it takes 13 min for completion [16, 17]. As the use of time consuming tools is not usually affordable in those settings, the use of short versions become crucial. In fact, short versions of the original FSFI scale have been proposed, which can be completed in 5 min or less[16, 17] and, indeed, used in Spain [19], which also reflects its clinical usefulness. However, to our knowledge, no FSFI short version has been validated in the Spanish population. Thus, our aim was to develop and validate a Spanish short version of the FSFI.

## Materials and methods

The development and validation of a Spanish short version of the FSFI was conducted through a parallel exploratory, sequential mixed-methods approach involving two sites (Fundació Puigvert, Barcelona and RAPbarcelona Clinical Centre, Barcelona). The process consisted of two steps: (1) cognitive and content validation, both through a focus group, and through item selection based on item response theory (IRT); (2) assessment of test–retest reliability.

### Cognitive and content validation of the FSFI

The FSFI comprises 19 items distributed in six domains: desire (items 1,2), arousal (items 3–6), lubrication (items 7–10), orgasm (items 11–13), satisfaction (items 14–16), and pain (items 17–19). This yields a final score of 2–36, with higher scores indicating more optimal sexual functioning [7, 8]. As no FSFI versions validated in the Spanish population were available at the time when the study was started, we conducted cognitive validation of the Spanish version of the FSFI translated by Blümel et al. [11]. This version was chosen because (a) the authors reported a correct backward translation technique to obtain the Spanish translation from the original English version and (b) this version was already in common use in our routine clinical practice and a priori women showed a good comprehension of all the items.

Cognitive validation of the FSFI was carried out by means of the focus group technique. This type of validation ensures correct understanding of questions and instructions by participants, as well as the identification of words and categories used by the target population, which can help in the reformulation of items [19]. The focus group was held in July 2018, and participants were approached through a convenience sampling method in the participating departments.

In both sites, the chiefs of the department were first approached via email in which the study was introduced and the specific collaboration they were asked for was described. The clinical centers were requested to select adults women with different social and academic profiles in order to ensure a greater range of inputs to our discussion. The stakeholders signed the informed consent to participate in the focus groups, with a final sample of 10 women aged older than 18. Final group was balanced in terms of age.

The focus group was carried out at the Fundació Puigvert meeting room and took approximately 3 h to complete. Three study investigators attend the meeting according to the focus group methodology.

At the beginning of each session, participants were provided with an explanation of the research and were encouraged to participate and give their opinion as much as possible. They were handed out a copy of the questionnaire and were asked to give a general opinion of the comprehension of the document, as well as rephrase complex questions in their own words. Beyond these, other specific questions regarding the most controversial points of the questionnaire were asked. At every participant's intervention, consensus was sought from all the members of the group. This meeting was recorded to allow its better transcription. Afterwards, the three investigators elaborated a final Spanish 19-item FSFI (sFSFI) version based on the analysis of the comments made by patients.

### Item selection by IRT analysis to elaborate the FSFI short version

A total of 114 consecutive female patients attending the outpatient clinics of the three participating departments were asked to complete the sFSFI. The needed number of participants was calculated taking into account the formulas developed by Jiang et al. [20]. The following patients were excluded: patients under 18 years old, patients who could not read, native Spanish speakers from countries other than Spain, patients with cognitive impairment or in whom, according to the investigators’ judgment, there was a risk of impaired interpretation of a scale, and patients who did not consent to enter the study.

After descriptive analysis of sample demographics and scale scores, item response theory (IRT) was the main analysis method. The IRT analyses were model-based, estimating the probability of item responses as a function of the level of the underlying construct being measured [21]. Analyses were conducted with IRTPRO 2.0 (Scientific Software International, 2013). The main aim of the IRT analyses was to create a short form of the FSFI. The IRT analyses included: (a) fitting an appropriate IRT model (the graded response model) to the ordinal-level data capturing participant responses to each item; (b) calibrating the items to obtain item difficulty parameters (represented by “b_i_” on Table 2, these parameters show what level of a trait or construct provided the most lower or better information in measuring), item discrimination parameters (represented by “a” on Table 2, this parameters which items provides more discrimination among participants and how accurately an item measures the underlying construct at its difficulty level), and item information estimates [22], and (c) identifying the subset of items that simultaneously maximized the scale’s measurement and included at least one item of each domain to cover the construct.

The result of this phase was the Spanish short version of the FSFI (sFSFI-sv). Scoring of each item was kept the same as in the original FSFI.

### Test–retest reliability of the Spanish FSFI short version

A sample of consecutive female patients attending the previous outpatient clinics was collected. Exclusion criteria were the same as in the previous phase. Patients were asked to complete the sFSFI-sv during the first visit (test). After 15 days, patients reporting no relevant clinical changes (measured by the Patient Global Impression of Improvement scale) were asked to attend a second visit to complete the sFSFI-sv again (retest).

The sample size was calculated using the formulas developed by Zou et al. [23] in concordance studies. An expected reliability was considered ρ_0_ = 0.850 and a minimum reliability was considered ρ_1_ = 0.750, with a statistical significance of 5% and a statistical power of 80%. Considering a missing data rate of 15%, the sample size necessary was 92 patients.

The statistical program IBM® SPSS® v.23.0 (IBM Corp., Armonk, NY, USA) was used for data analysis. A descriptive analysis of the sample and scale results was performed. Test–retest reliability was assessed with the intraclass correlation coefficient (ICC) or kappa index for scales' total score or item scores respectively (γ). Excellent reliability was considered present when γ > 0.8, good reliability when γ > 0.6, and moderate reliability when γ > 0.4 [24].

### Spanish FSFI short version cut off calculation

We calculated a cutoff point of sFSFI-sv in order to establish the total score of the sFSFI-sv that could indicate presence or absence of sexual disorder. Based on clinical interview, the same sample of patients were also classified according to the presence or absence of a sexual disorder (either orgasmic, of interest/arousal or genito-pelvic pain/penetration disorder), following the Diagnostic and Statistical Manual of Mental Disorders, fifth edition (DSM-V) criteria [25].We used the presence or absence of a sexual disorder variable to calculate the total score point of the sFSFI-sv whose Area Under the Curve (AUC) based on sensibility and specificity was maximum on the Receiver Operating Characteristics (ROC).

## Results

### Cognitive and content validation of the FSFI

After an exhaustive reading of the Spanish FSFI scale, comments were collated regarding grammar, spelling, and any expressions that were different from South American Spanish and on this basis appropriate modifications were made. Additionally, some concerns were raised regarding conceptual aspects of the scale, the most relevant being that two out of three satisfaction domain items referred to a partner relationship, while satisfaction with sexual activity may be excellent in non-partner relationships. However, the decision was taken not to implement modifications relating to such concerns since they would have altered the meaning of the items and consequently of the whole scale.

### Elaboration of the Spanish FSFI short version

One hundred and fourteen women were included, with a mean age of 53.5 (14.9) years. Their demographic characteristics are shown in Table 1.

**Table 1:** Characteristics of patients included in the study

	sFSFI-sv elaboration	Test–retest reliability and criterion validity
All patients	114	93
Age (years), mean (SD)	53.5 (14.9)	54.9 (13.9)
*Education, N (%)*		
None	2 (1.8)	2 (2.2)
Elementary	21 (18.4)	18 (19.4)
Secondary	34 (29.8)	26 (28)
Higher	57 (50)	47 (50.5)
*Marital status, N (%)*		
Single	5 (4.4)	5 (5.4)
Married or steady partner	96 (84.2)	76 (81.7)
Divorced	7 (6.1)	7 (7.5)
Widow	5 (4.4)	5 (5.4)
Children, N (%)	90 (78.9)	74 (79.6)
Menopausal, N (%)	70 (61.4)	57 (61.3)
Hormone replacement therapy, N (%)	5 (4.4)	4 (4.3)
*Reason for referral, N (%)*		
Incontinence	46 (40.3)	39 (41.9)
Pelvic organ prolapse	13 (11.4)	12 (12.9)
Pelvic floor muscle dysfunction	5 (4.4)	4 (4.3)
Pelvic pain	10 (8.7)	7 (7.5)
Sexual dysfunction	19 (16.7)	15 (16.1)
Others	21 (18.4)	16 (17.2)
*Comorbidities, N (%)*		
Oncologic disease	8 (7)	7 (7.5)
Neurologic disease	4(3.5)	4 (4.3)
Depression	19 (16.7)	16 (17.2)
Anxiety	11 (9.6)	9 (9.7)
Prior pelvic surgery	35 (30.7)	29 (31.1)
*Frequency of sexual activity, N (%)*		
> 4 per week	1 (0.9)	0 (0)
3–4 per week	3 (2.6)	3 (3.2)
1–2 per week	33 (28.9)	22 (23.7)
1–2 per month	42 (36.8)	36 (38.7)
< once per month	35 (30.7)	32 (34.4)

Continuous variables are expressed as mean (SD), while non-continuous variables are expressed as N (%). sFSFI-sv: Spanish Female Sexual Function Index – short version

The initial IRT model using all 19 items of the FSFI resulted in a significant S-χ^2^ for items 1, 2, 5, 11, 18, and 19 (all p < 0.001 with Bonferroni-corrected alpha of 0.003). The likelihood-based Goodness of Fit Statistics were Akaike information criterion (AIC) 4468.13 and Bayesian information criterion (BIC) 4758.78.

As discarding items 1 and 2 would have meant no representation of the desire domain, and it was the decision of the investigators to maintain all domains in the final scale, the focus group comments were taken into account and on this basis item 1 was selected for inclusion in the final scale.

Subsequent IRT analysis was carried out using the 14 items selected, and a non-significant S-χ^2^ (all p > 0.001 with Bonferroni-corrected alpha of 0.003) was found for all items. The likelihood-based goodness of fit statistics were AIC 3264.62 and BIC 3480.02, respectively. Both were smaller than in the initial model. Accordingly, the 14-item model adjusted better. The χ^2^-Pearson test of the likelihood ratio between the two models was statistically significant (p < 0.001).

Table 2 shows the difficulty and discrimination parameter estimates and their standard error for the 14 remaining items of the FSFI.

**Table 2:** Difficulty (b) and discrimination (a) parameter estimates and their standard error for the 14 remaining items of the FSFI

Domain	Item	*a*	*s.e*	*b*_1_	*s.e*	*b*_2_	*s.e*	*b*_3_	*s.e*	*b*_4_	*s.e*	*b*_5_	*s.e*
Desire	1	1.11	0.30	− 1.70	0.54	− 0.33	0.25	1.02	0.26	3.57	1.00		
Arousal	3*	5.88	1.17	− 1.10	0.25	− 0.66	0.20	− 0.28	0.13	0.27	0.11	0.79	0.18
	4	4.13	1.08	− 1.04	0.19	− 0.66	0.16	− 0.13	0.11	0.59	0.14	1.42	0.22
	6	4.50	1.30	− 0.84	0.14	− 0.43	0.10	− 0.21	0.09	0.25	0.09	0.79	0.18
Lubrication	7	8.03	3.27	− 0.90	0.32	− 0.27	0.12	0.00	0.06	0.28	0.10	0.76	0.26
	8	5.04	1.69	− 0.90	0.27	− 0.63	0.21	− 0.22	0.08	0.22	–	0.96	0.24
	9*	8.64	3.10	− 0.79	0.25	− 0.31	0.10	0.07	0.06	0.38	0.05	0.76	0.25
	10	4.37	1.98	− 0.92	0.41	− 0.71	0.35	− 0.27	–	0.12	–	0.90	0.47
Orgasm	12*	3.77	0.85	− 1.02	0.20	− 0.44	0.14	− 0.20	0.10	0.17	0.14	0.99	0.14
	13	3.58	0.96	− 0.86	0.19	− 0.24	0.13	− 0.08	0.12	0.12	0.14	0.79	0.14
Satisfaction	14*	2.18	0.28	− 0.68	0.14	− 0.57	0.15	− 0.43	–	− 0.34	–	0.43	–
	15	1.74	0.35	− 1.97	0.44	− 1.11	0.38	− 0.70	0.26	− 0.09	0.10	1.21	0.32
	16	1.82	0.47	− 1.75	0.46	− 0.97	0.32	− 0.51	0.38	0.00	–	1.44	0.35
Pain	17	3.17	0.58	− 0.50	0.01	− 0.05	0.13	0.10	–	0.27	0.11	0.55	–

^*^ Items with highest discrimination and optimal difficulty indexes

On the basis of the results of the discrimination and difficulty analysis, item 3 of the arousal domain, item 9 of the lubrication domain, item 12 of the orgasm domain, and item 14 of the satisfaction domain should have been included in the final scale. Concordance with focus group content validity assessment was observed for items 3, 9, and 12. Regarding satisfaction, item 16 was preferred by the focus group participants, and it was the decision of the investigators to include it in the final scale in preference to item 14. Ultimately a six-item unidimensional scale was agreed as the final version (items 1, 3, 9, 12, 16, and 17) (Table 3). As each item was scored as in the original FSFI from 0 or 1 to 5, the range of this short form was from 2 to 30.

**Table 3:** Final Spanish short version of the Female Sexual Function Index

Original FSFI Item number	Spanish final translation after cognitive and content validation and scoring
1	*En las últimas 4 semanas, ¿Con qué frecuencia ha sentido deseo o interés sexual?* (Over the past 4 weeks, how often did you feel sexual desire or interest?) * Siempre o casi siempre* 5 (Almost always or always) * Bastantes veces* 4 (Most times (more than half the time)) * A veces* 3 (Sometimes (about half the time)) * Pocas veces* 2 (A few times (less than half the time)) * Casi nunca o nunca* 1 (Almost never or never)
3	*En las últimas 4 semanas, ¿Con qué frecuencia ha sentido excitación sexual durante la actividad sexual?* (Over the past 4 weeks, how often did you feel sexually aroused ("turned on") during sexual activity or intercourse?) * No he tenido actividad sexual* 0 (No sexual activity) * Siempre o casi siempre* 5 (Almost always or always) * La mayoría de las veces (más de la mitad)* 4 (Most times (more than half the time)) * A veces (alrededor de la mitad)* 3 (Sometimes (about half the time)) * Pocas veces (menos de la mitad)* 2 (A few times (less than half the time)) *Casi nunca o nunca* 1 (Almost never or never)
9	*En las últimas 4 semanas, ¿Con qué frecuencia se ha sentido lubricada (humedad vaginal) hasta el final de la actividad sexual?* (Over the past 4 weeks, how often did you maintain your lubrication ("wetness") until completion of sexual activity or intercourse?) * No he tenido actividad sexual* 0 (No sexual activity) * Siempre o casi siempre* 5 (Almost always or always) * La mayoría de las veces (más de la mitad)* 4 (Most times (more than half the time)) * A veces (alrededor de la mitad)* 3 (Sometimes (about half the time)) * Pocas veces (menos de la mitad)* 2 (A few times (less than half the time)) * Casi nunca o nunca* 1 (Almost never or never)
12	*En las últimas 4 semanas, cuando ha tenido estimulación sexual y/o relaciones sexuales, ¿Le ha sido difícil alcanzar el orgasmo o clímax?* (Over the past 4 weeks, when you had sexual stimulation or intercourse, how difficult was it for you to reach orgasm (climax)?) * No he tenido actividad sexual* 0 (No sexual activity) * Extremadamente difícil o imposible* 1 (Extremely difficult or impossible) * Muy difícil* 2 (Very difficult) * Difícil* 3 (Difficult) * Poco difícil* 4 (Slightly difficult) * Nada difícil (fácil)* 5 (Not difficult)
16	*En las últimas 4 semanas, ¿Cómo de satisfecha ha estado con su vida sexual en general?* (Over the past 4 weeks, how satisfied have you been with your overall sexual life?) * Muy satisfecha* 5 (Very satisfied) * Moderadamente satisfecha* 4 (Moderately satisfied) * Ni satisfecha ni insatisfecha* 3 (About equally satisfied and dissatisfied) * Moderadamente insatisfecha* 2 (Moderately dissatisfied) *Muy insatisfecha* 1 (Very dissatisfied)
17	*En las últimas 4 semanas, ¿Con qué frecuencia ha sentido molestia y/o dolor durante la penetración vaginal?* (Over the past 4 weeks, how often did you experience discomfort or pain during vaginal penetration?) * No he tenido actividad sexual* 0 (Did not attempt intercourse) * Siempre o casi siempre* 1 (Almost always or always) * La mayoría de las veces (más de la mitad)* 2 (Most times (more than half the time)) * A veces (alrededor de la mitad)* 3 (Sometimes (about half the time)) * Pocas veces (menos de la mitad)* 4 (A few times (less than half the time)) * Casi nunca o nunca* 5 (Almost never or never)

The final score is the sum of the ordinal responses to the six items; the score can range from 2 to 30

### Test–retest reliability of the Spanish FSFI short version

Ninety-three women were included, with a mean age of 54.9 (13.9) years. All completed both the test and the retest sFSFI-sv. Their characteristics are shown in Table 1.

The kappa coefficient showed moderate reliability for desire (0.72), arousal (0.69), lubrication (0.71), orgasm (0.71), and satisfaction (0.74) and good reliability for pain (0.80). However, the intraclass correlation coefficient showed excellent reliability for the total score of the sFSFI-sv (0.91).

### Spanish FSFI short version cut off calculation

**Fig. 1:**
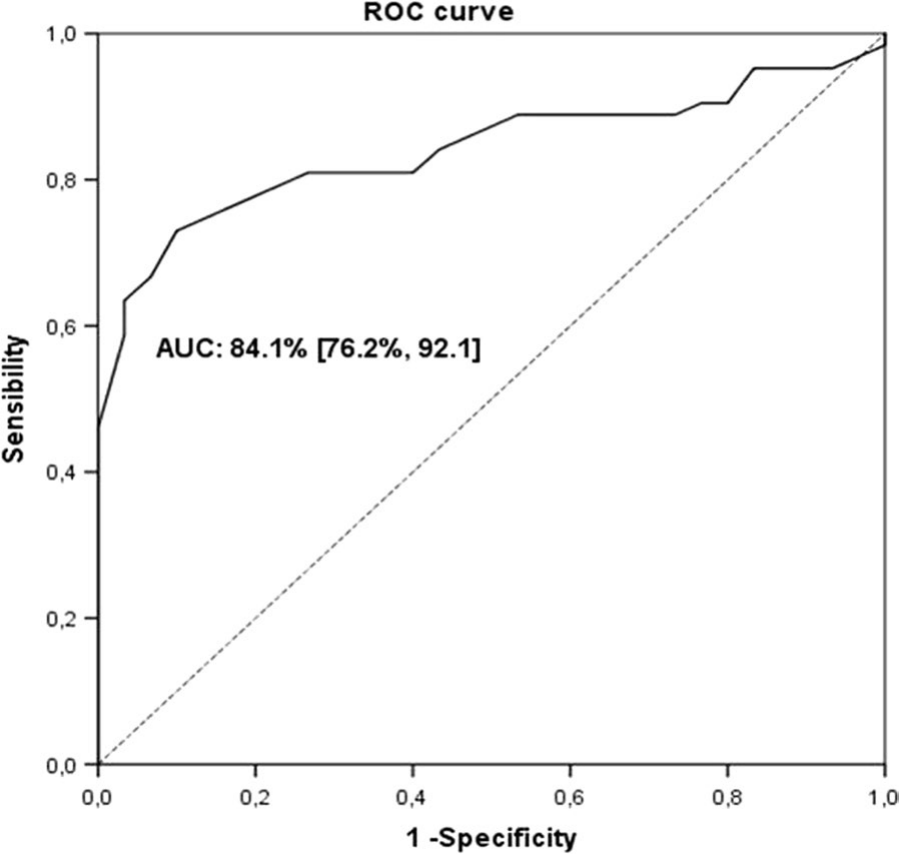
Receiver Operating Characteristics (ROC) curve of the Spanish FSFI short version to establish a cut off score to detect women at higher risk of sexual disorder

Mean results of sFSFI-sv was 19.2 (7.3). Thirty women (32.3%) presented a sexual disorder based on clinical interview, while 63 women (67.7%) did not present a sexual disorder. The AUC was 84.1% with a CI95% [76.2%, 92.1%]. A total score equal or less than 18 on the sFSFI-sv would indicate that the participant is at higher risk of sexual disorder, with a 81.0% of sensitivity and a 73.3% of specificity (Fig. 1). Seventy three of the 93 participants were classified as true positive or negative.

## Discussion

In this study we conducted a cognitive validation of the FSFI scale in a Spanish population using focus group methodology, developed its short form through item response theory (IRT) for item selection, and proceeded to assess its reliability in a sample of Spanish female patients. Specific modifications of the FSFI were made according to the focus group results. Following the IRT analysis, FSFI items 1, 3, 9, 12, 16, and 17 were selected for inclusion in the final Spanish FSFI short version (sFSFI-sv), which showed good reliability.

All domains of the original FSFI scale were included in the scale derived through our analysis, as is also true for the FSFI short forms proposed by Carpenter et al. [16] and Isidori et al. [17]. According to the initial IRT model using all 19 items, the entire desire domain should have been excluded from the final scale. Since in DSM-V disorders of interest and arousal are included in the same diagnostic category [25], mainly due to the overlap between the two phases of sexual response and ensuing symptoms, exclusion of the desire domain could have been justified by maintaining the arousal domain to identify patients of this diagnostic category. In fact, the authors of the original FSFI stated that a five-domain scale combining interest and arousal would also have been feasible [26]. However, like the authors of the original scale [26], we preferred to keep the six-domain structure and ultimately included the desire item in the final version of the short form.

When analyzing the items selected for the final sFSFI-sv, we realized that there was not full agreement with other authors’ selections [16, 17]. Carpenter et al. [16] supported by their IRT analysis, tended to include items related to severity and difficulty rather than frequency items. In contrast, in our IRT analysis, frequency items consistently showed better discrimination and difficulty parameters, which explained the items selected for the arousal, lubrication, and orgasm domains. This could be explained by linguistic and cultural factors, as within the focus group items exploring grade, intensity, or severity of the symptoms were those that generated greater confusion and discussion. This fact led us to think that, beyond a proper backward translation technique, transcultural cognitive validation of scales is of crucial relevance in psychometric validation processes. Following the same argumentation, and according to the focus group comments, we chose item 1 and not item 2 to represent the desire domain in the final sFSFI-sv.

Regarding the satisfaction domain, IRT analysis identified item 14 as that with the best discrimination and difficulty parameters. Although items related to sexual activity involving a partner gave rise to no difficulties in terms of linguistic comprehension within the focus group, there was a general consensus among patients that satisfaction with sexual activity could be high in the absence of a steady partner. Moreover, although we acknowledge that marital status can be related to sexual satisfaction [28], including such an item in the final sFSFI-sv version would prevent women without a steady partner from filling in the item and consequently from completing the whole scale. Thus, item 16 was preferred for inclusion in the final sFSFI-sv version. No conflicts were detected between the focus group comments and the IRT analysis regarding selection of the pain item, which is in line with that selected by other authors [16, 17].

According to our results, in a range from 2 to 30, women with higher sFSFI-sv scores could be at lower risk of suffering from sexual dysfunction, while scores equal or less than 18 could indicate the presence of a sexual disorder. However, we have to take into account the fact that the FSFI, and consequently the sFSFI-sv, cannot be considered a diagnostic tool as it does not measure distress of these patients, which is a requirement for the diagnosis of sexual dysfunction [25]. Thus, although it seems a practical tool for screening women in clinical practice, it cannot replace other diagnostic tools or clinical judgment.

The main advantage of the sFSFI-sv is the less time needed for its completion and interpretation, while assessing all the female sexual function domains assessed in the original questionnaire and without losing relevant information. However, the original FSFI questionnaire could be useful when one aims to deepen the qualitative analysis of a particular aspect of female sexual function.

Our study has some limitations. First, we did not perform backward translation. We considered the methodology described by Blümel et al. [11] as appropriate, and the resulting scale, commonly used in our setting, underwent a cognitive transcultural validation process to detect any possible misunderstandings between Chilean and Spanish populations. Secondly, as previously stated, we can't consider the sFSFI-sv as a diagnostic tool for sexual dysfunction as it does not assess distress of these patients. To our knowledge, this is the first Spanish validation of an FSFI short version, conducted through a structured methodology. Thus, the sFSFI-sv could be used to facilitate and spread the assessment of sexual function among Spanish women either in routine clinical practice or in a research setting.

## Conclusion

After cognitive and content FSFI validation of the Spanish FSFI, the IRT analysis and the focus group methodology allowed the development of a six-item Spanish version of the FSFI which showed good reliability in a group of Spanish women. This scale could be used to facilitate the assessment of female sexual function.

## Data Availability

The datasets used and/or analyzed during the current study are not publicly available due to personal data protection policy, but are available from the corresponding author on reasonable request.

## Abbreviations

DSM-V: Diagnostic and Statistical Manual of Mental Disorders, fifth edition
FSD: Female Sexual Dysfunction
FSFI: Female Sexual Function Index
ICC: Intraclass correlation coefficient
IRT: Item response theory
sFSFI-sv: Spanish Female Sexual Function Index- short version
